# Assembly Theory: What It Does and What It Does Not Do

**DOI:** 10.1007/s00239-024-10163-2

**Published:** 2024-03-07

**Authors:** Johannes Jaeger

**Affiliations:** 1 Department of Philosophy, University of Vienna, Universitätsstraße 7, 1010 Vienna, Austria; 2 Complexity Science Hub (CSH) Vienna, Josefstädter Straße 39, 1080 Vienna, Austria; 3 Ronin Institute, Montclair, USA

**Keywords:** Assembly theory, Complexification, Evolutionary innovation, Natural selection, Darwinian evolution

## Abstract

A recent publication in *Nature* has generated much heated discussion about evolution, its tendency towards increasing diversity and complexity, and its potential status above and beyond the known laws of fundamental physics. The argument at the heart of this controversy concerns assembly theory, a method to detect and quantify the influence of higher-level emergent causal constraints in computational worlds made of basic objects and their combinations. In this short essay, I briefly review the theory, its basic principles and potential applications. I then go on to critically examine its authors’ assertions, concluding that assembly theory has merit but is not nearly as novel or revolutionary as claimed. It certainly does not provide any new explanation of biological evolution or natural selection, or a new grounding of biology in physics. In this regard, the presentation of the paper is starkly distorted by hype, which may explain some of the outrage it created.

## A commotion

There has been a lot of agitation over the past few weeks about a paper that recently appeared in *Nature* (Sharma et al. 2023; see also the news & views feature by Ellis 2023)*.* Many of my fellow evolutionary biologists felt compelled to express their disgust and outrage on social media. Sadly, there is much shouting but little serious argumentation on display.^1^ Evolutionary biology is being grossly misrepresented, they say. Even worse: the perpetrators are chemists and physicists who are claiming that there is an explanatory gap between physics and evolution. Accusations of “nonsense,” “mumbo jumbo,” “word salad,” creationist intent, and (perhaps most surprising of all) injecting wokeness into evolutionary theory soon started flying from all kinds of directions.

But what is all the fuss about? It’s about something called *assembly theory*, which was created by Sara Walker, Lee Cronin, and colleagues*.* It has been around for a few years (Marshall et al. 2017, 2021, 2022; Liu et al. 2021), and has ruffled some feathers previously within the communities of combinatorial chemistry^2^ and complexity science^3^ (Uthamacumaran et al. 2022). So what exactly is going on?

To get straight to the point: *I find assembly theory intriguing and worth considering*. Originally developed to detect signatures of alien life in the atmosphere of faraway exoplanets^4^ (Marshall et al. 2017, 2021), it is a neat and simple model for the combinatorial generation of innovation in rule-based abstract “worlds” of recombining objects. Arguably, it is a prime example of classic computational complexity theory. Applied to the natural world, the metrics it proposes may allow us to detect whether levels of organization above the basic laws of physics have emerged in an observed system, and to estimate the causal influence of these higher levels on the underlying dynamics. As an added bonus, assembly theory manages all this without having to assume anything specific about what those higher levels of organization actually *are*. To me, this sounds interesting and potentially useful. We should not dismiss the work outright and, despite our skepticism, give the theory a fair chance to prove its worth.

On the downside, there are several serious problems that stem from the framing and presentation of the argument in the paper, the hype the authors have generated around it (online and in peer-reviewed print), and their problematic and overextended interpretation of the model.

The first major issue is that the paper is entitled “*Assembly theory explains and quantifies selection and evolution*” (Sharma et al. 2023) when it does absolutely no such thing. In fact, assembly theory is not specifically about Darwinian evolution by natural selection and, besides, the paper uses the term “selection” in a very broad sense that no biologist would recognize. This leads to unnecessary confusion, as is evident from the angry online comments. To make things worse, the abstract is a textbook example of how *not* to write an abstract. It is phrased in vague and hyperbolic terms that are misleading. We will come back to that later.

At this point, let me make one point very clear: it is not fair or proper to dismiss a paper based on its abstract alone, which is exactly what a surprising number of online commenters did who obviously (and sometimes admittedly) did not bother to read the whole paper.

That’s why I *did* read the paper carefully (plus some of its predecessors and criticisms as well). This helped me clarify what assembly theory is good for, and what its limitations are. Let me share these insights with you, and then quickly review the entire controversy within the context of contemporary scientific publishing and career structures.

## Some Assembly Required

**Fig. 1:**
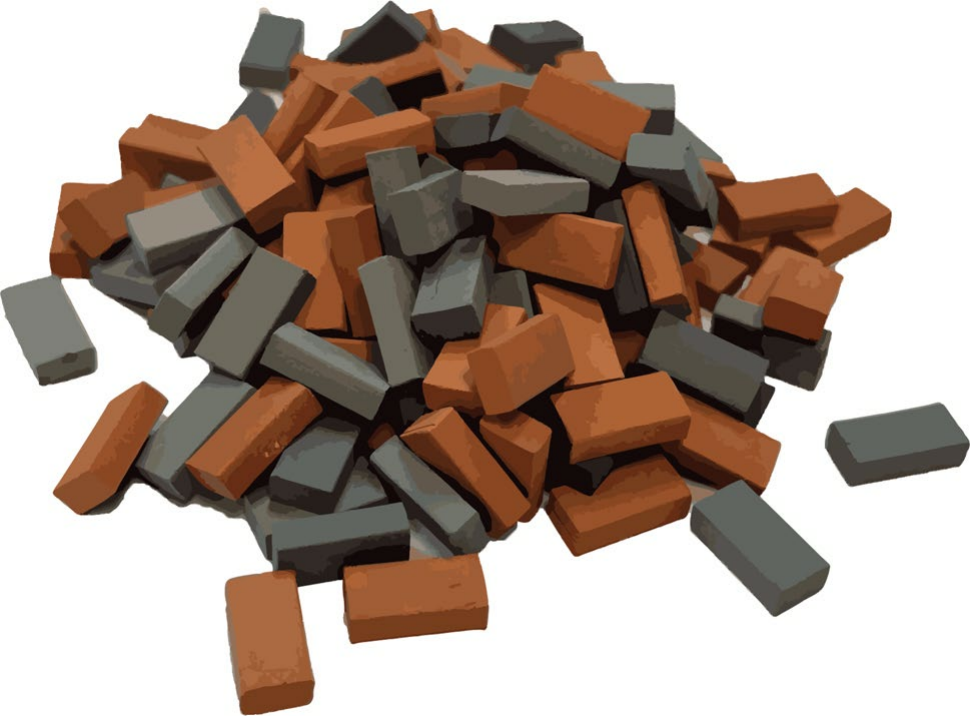
Building blocks of an assembly universe

The fundamental idea behind constructing an assembly theory model is that you define an “*assembly universe*,” which consists of a finite set of distinct basic building blocks (colored bricks in Fig. 1) and another finite set of rules that allow you to assemble them into more complex composite objects. This universe, in principle, can contain all possible combinations of building blocks that do not violate your basic assembly rules. If you want to simulate real-world chemistry, for example, you can derive all kinds of composite molecules by recombining atoms with their corresponding chemical bonds.

**Fig. 2:**
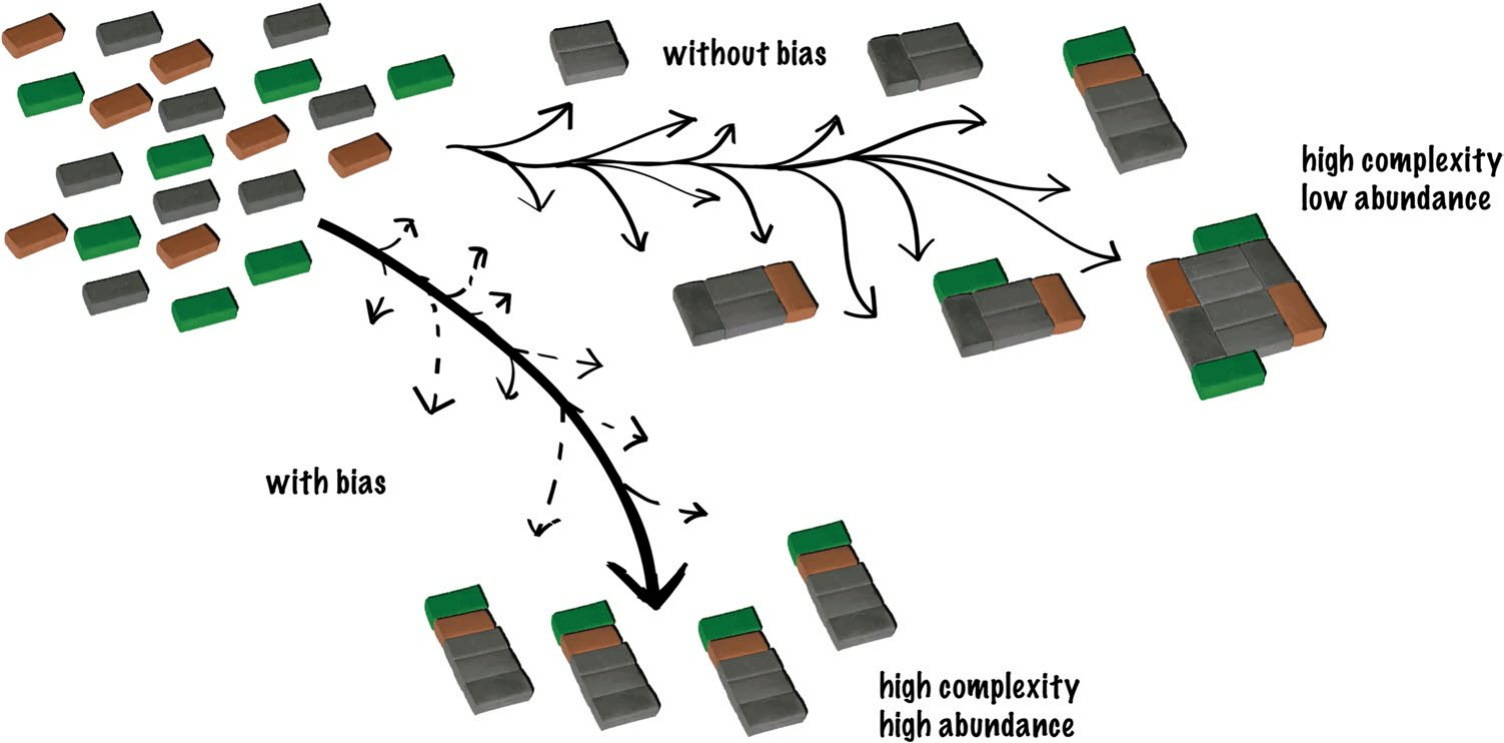
An assembly process (adapted from Sharma et al. 2023)

So far so good. Now you introduce a dimension of time to the model, which is implemented by *recursivity*. In other words, at each step of the assembly process, you can use all objects that are already assembled (not just the basic building blocks you started with) for further assembly. Thus, at each step, you get a bigger choice of objects to build with. In fact, the number of possible rule-based combinations will increase hyper-exponentially with each step. This way, you can see what kinds of composites emerge over time. An example of such a process is depicted in Fig. 2.

Recursivity makes the dynamics of the model *historically contingent*. In the end, the kinds of objects that you actually *can* assemble are not only restricted by the rules of your universe, but also by the starting point and trajectory you actually chose to take. This renders the whole model computationally tractable, because it greatly reduces the combinations of possible composites you can get. You don’t have to deal with all possible combinations of building blocks, just the ones that are actually present in whatever “world” (a mix or “ensemble” of different objects) that you are simulating or observing. For instance, you can start with a given set of substrates, and simulate what kind of products you can get from there, given all possible chemical reactions plus the relative abundances of the substrates.

You can consider such a system a kind of a null model: given your basic building blocks and the assembly rules you defined, you can calculate how many steps you need to take before a specific composite object can arise. That minimal path to construct a composite object is called its *assembly index*. If your rules have specific weights or probabilities of application, you can also predict the expected abundance (copy number) for any composite object in your mix. This may not be easy to do in practice, but the authors at least show that it is possible in principle for any well-defined world with finite sets of building blocks and rules.

By the way, these metrics are not particularly novel. They are special cases of classical measures of *algorithmic* or *computational complexity* (especially Huffman 1952; Bennett 1988), which are used in a peculiar combination here, tailored to the context of assembly theory. We will come back to this point later.

**Fig. 3:**
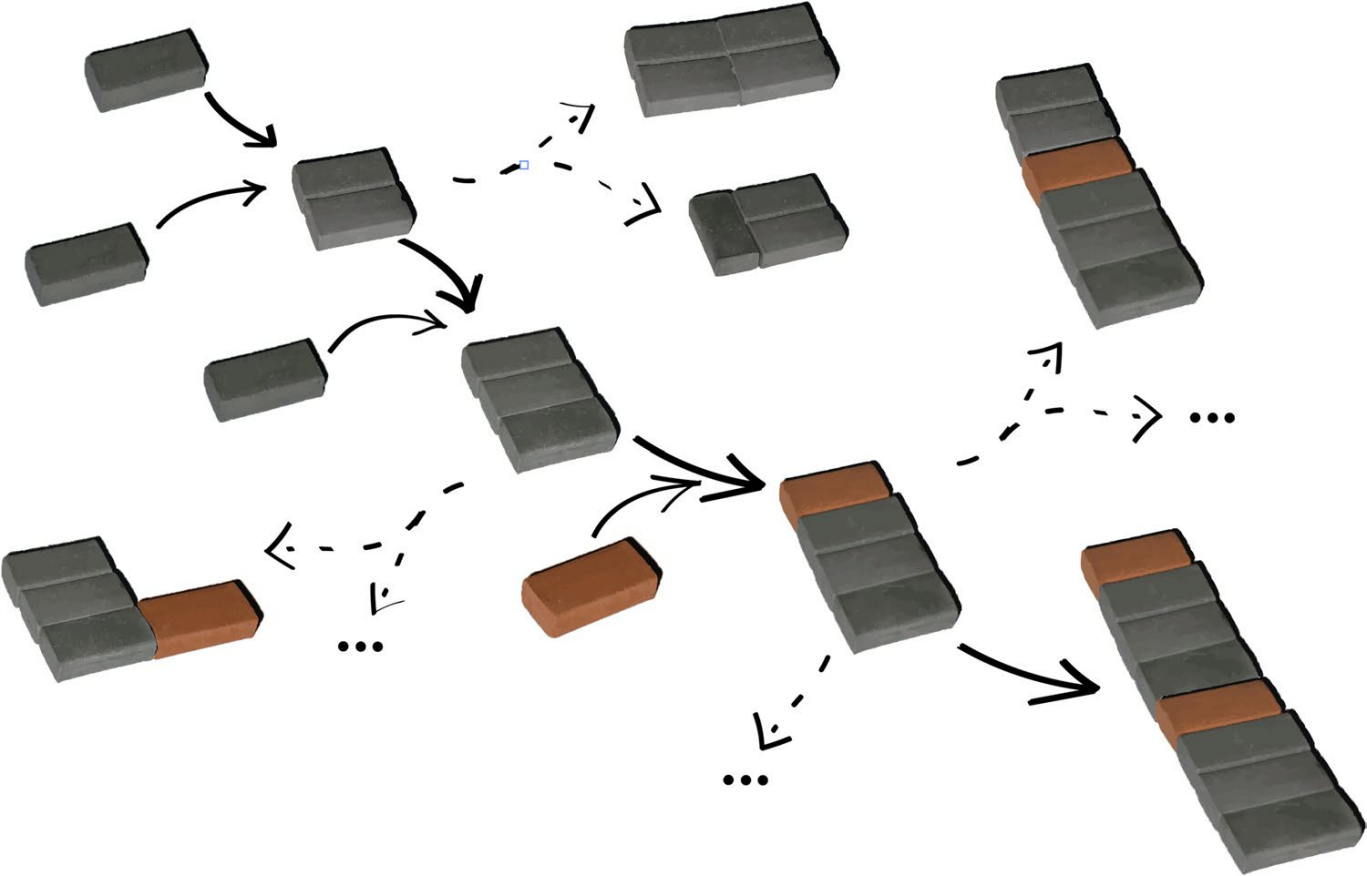
“Selection” in assembly theory means bias in outcome (adapted from Sharma et al. 2023)

Things become truly interesting once your model produces very complex composites. The higher the complexity of a composite, the longer it will take to appear, and the more unlikely it is to appear by chance, especially after just a minimal number of steps. Based on this, you would generally expect many different complex composites to be present at very low abundance at later steps. Yet, *if you find certain complex composites enriched, especially early on, that’s a sign that things are not just based on the random interplay of the basic rules in your system*. Figure 3 illustrates the point.

Put simply: finding composites with high complexity at high abundance means the basic rules of your “world” have probably been skewed in some way that is not built into the basic rules. That’s what the authors mean by “*selection*” (Fig. 3). This concept is, of course, a whole lot broader and more generic than what evolutionary biologists mean by the term. And the bias that assembly theory calls “selection” could be caused by processes that are very different from Darwinian evolution (many of them not selective at all). We’ll come back to that shortly. For now, let me reiterate that “selection” in the context of assembly theory simply means that the basic rules of the world you have defined have been constrained or channeled in some way to lead to an unexpected outcome.

This conception of bias is not mysterious at all. It simply means that *you can use assembly theory to check whether something unexpected is going on* in a very broad range of computational model “worlds” or “universes” defined by different building blocks and rules. If that “something” is present, then more than just your basic rules must be at work. As a practical example, the authors suggest that assembly theory can be used to analyze mass spectrographs of exoplanet atmospheres in search for complex molecules at high abundance, which would mean something more than just the laws of chemistry are at work there (Marshall et al. 2017, 2021). Likewise, you could monitor the complexity and abundance of some technology, let’s say Schnitzel hammers^5^ in Austria, to infer that this technology must have been selected for in a particular environment. It did not just pop up randomly, but co-evolves with its substrate, the Viennese Schnitzel. So far, so good.

At this point it’s important to reiterate that *assembly theory does not (and need not) make any assumptions as to what is being “selected,” and in what way*. On the one hand, this is bad: it is one of the things that is confusing to evolutionary biologists complaining about the model. Assembly theory is not specifically about Darwinian evolution. It does not care about populations, individuals, genes, and so on. It does not fit into the conceptual framework of traditional evolutionary theory, that is for sure. And it almost certainly won’t help you to measure selective pressures in the wild. On the other hand, its lack of specificity is also a strength of assembly theory: it is very broadly applicable to all kinds of “worlds” and “selection” biases (if you can formulate an appropriate “world” and figure out how to measure the assembly index, which is far from trivial in most cases).

In other words, *assembly theory is a tool to detect the emergence of new levels of organization and their causal influence on lower-level phenomena in the world you are observing*. If the outcome you are detecting is biased, the underlying rules must have been constrained or channeled in some way to generate that bias. Philosophers call this “*downward causation*,” and keep on arguing about it. In particular, some reductionists find it objectionable, but for reasons that really don’t hold up to closer scrutiny. To explain why would take another paper-length argument. Suffice it to say that the problem goes away if you consider that processes and relations (*i.e.*, the rules that are affecting your objects) are fundamental, and not only the objects with their intrinsic properties themselves.

All you need to know about downward causation in this context is that it does not change the underlying rules. Instead, it constrains and channels the underlying processes in unexpected ways. And, if you think about it, that’s exactly what evolution does with the laws of physics: natural selection never alters the rules of physics and chemistry underlying the processes that compose your body, but constrains and channels the direction of these processes in ways that you fundamentally cannot predict from the underlying physics or chemistry alone. This is why (evolutionary) biology is (and will always remain) an independent science after all. And in this sense, there undoubtedly *is* an explanatory gap between physics and biology, as the authors rightly point out.

It is in this very general and roundabout way that *assembly theory can indeed be useful for evolutionary biology*. It may allow us to establish, once and for all, that biology *is* more than just chemistry and physics. More specifically, it provides us with a potentially useful perspective on what may be driving innovation and complexification in evolution (and other higher-level processes) at a very fundamental level: it’s the emergence of ever new combinations of (chemical and higher-level cell-, tissue-level, or organismic) components. It’s *the constant growth of a co-evolving possibility space*, something which biologist Stuart Kauffman (2000) has called *the adjacent possible*. This should make us reconsider how we formally describe what is possible in evolution, and it adds a temporal directionality to the random effects of mutations: for a mutation to produce a complex outcome, more time will be required than for simple ones.

Arguably, *this is not exactly rocket science* and assembly theory oversimplifies the complexity of real-world evolutionary innovation to the point of potentially losing its connection with reality altogether. Yet, I would say it *is* a step forward compared to accounts of evolution that simply take the emergence of complexity for granted or assume a global preexisting space of possibilities for evolution. Such views are outdated and unhelpful, but still surprisingly common among reductionist evolutionary biologists and traditional complexity scientists, and that *is* a problem, in my opinion.

**Fig. 4:**
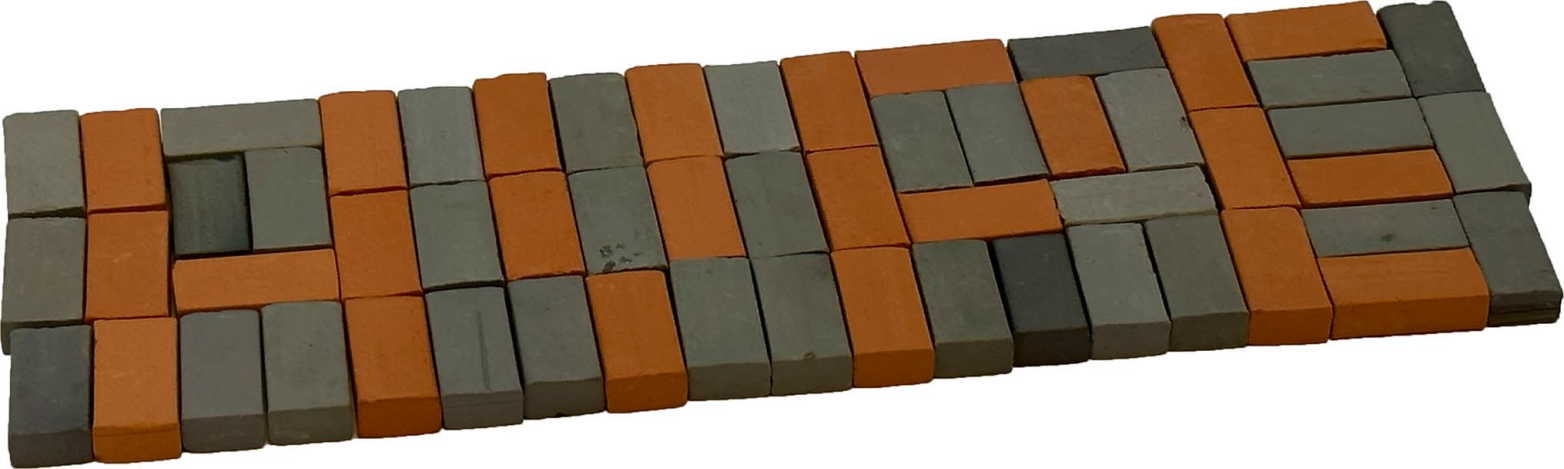
Assembly theory is surrounded by hype

For this reason, *it would definitely not hurt if some of the people disparaging assembly theory as a matter of principle would profit from engaging with it in a more good-faith and open-minded manner.* A new perspective, even if controversial and not yet thoroughly worked out, can be inspiring and useful. Give it some slack! Personally, I find it enriching to engage with a different point of view every once in a while, because it allows me to see the advantages and limitations of my own approach more clearly. Judging from what is going on online or when I get my papers or grants reviewed, this is not a common attitude in our field. And that is a real shame, in my opinion. It clearly limits the ways we think about the incredible richness of evolutionary phenomena. And this, I firmly believe, is hampering conceptual progress in our field right now (Fig. 4).

## A Flurry of Hype

Having said all this, it is time to turn to some of the issues surrounding the paper. The main problem is that it is wrapped in a misleading package, while much of the actual substance of the work is hidden in the technical appendices. Let me emphasize again that assembly theory is *not* a new theory of Darwinian evolution, nor is it a new interpretation of the concept of “selection” used in Darwinian theory. The title raises expectations the paper simply cannot meet. The authors do not even bother to explain how assembly theory could be applied in the context of genes, organisms, or populations. Granted, assembly theory *can* be applied to detect the signature of Darwinian evolution, but that requires all sorts of auxiliary hypotheses. For instance, the presence of specific chemical signatures in an exoplanet’s atmosphere only indicate the presence of alien life if we can confidently exclude potential sources for the detected bias other than biological evolution.

What are such alternative higher-level constraints? They can arise through self-organization, such as that observed in far-from-equilibrium (dissipative) systems, *e.g.*, hurricanes, eddies, and candle flames, which can form highly complex and improbable structures. It can also arise through the peculiar self-referential organization of living matter, which goes beyond mere self-organization in non-living systems (see Jaeger 2024a, 2024b, and references therein). Yet, deviations from expected outcomes can also occur for much more mundane reasons. For instance, kinetic energy barriers can heavily alter the rates of chemical reactions influencing what steady states will be reached. Furthermore, stochastic phenomena such as founder effects and random drift will play a role at early stages, especially in relatively small worlds. Or bias can be due to some hidden or neglected causal factor that was omitted from the basic rules when formulating the model.

Therefore, *how we interpret bias in outcomes largely depends on auxiliary hypotheses and the very assumptions we put into the model of our rule-based world in the first place*. This kind of circularity is not necessarily vicious, and is common in scientific modeling practice, but it clearly begs the question the paper is claiming to address. To say it again: assembly theory cannot tell us whether some bias is due to natural selection or not. It only tells us whether the bias is there, and how much of it is there, given the basic assumptions underlying the rule-based world we are modeling with assembly theory.

Of course, this also means that all the talk about *biological function* in the paper remains completely vacuous. If you cannot tell whether some bias in your outcome is the product of actual natural selection, you cannot tell whether it is functional in a sense of the term any biologist would recognize. As a matter of fact, *assembly theory does not deal with biological function at all.* So why do the authors bring it up?

Are these various blind spots of assembly theory known to the authors? I would strongly hope so. Otherwise, they would be rather fundamentally misinterpreting the nature and reach of their own model. I choose to give them the benefit of doubt, and will assume that they realize they are using the term “selection” in a broad and vague sense, including biases that are not selective at all. But if they realize this, how can they not see that their use of the term “selection” is grossly misleading?

Based on the evidence presented so far, I cannot help but conclude that we are dealing with a rather disingenuous presentation of what the paper actually achieves. *What assembly theory really does is to detect and quantify bias caused by higher-level constraints* in some well-defined rule-based worlds. That’s it! Even if Darwinian selection may contribute to such bias, assembly theory cannot tell you if it does, how much it does, or if other factors play a role as well.

Why this kind of packaging? It seems to do so much more harm than good. In fact, there is much food for thought hidden behind a thick smokescreen of impenetrable language and implausible claims. But it is probably exactly the glitzy packaging that got the paper into *Nature* in the first place. This highlights well-known weaknesses and failures in the editorial and peer-review processes of high-profile scientific journals. Exposure in high-profile journals still counts for far too much social capital in today’s scientific career and funding market, and selling assembly theory for what it really is was evidently not sexy enough to get that kind of exposure.

All this reflects rather unfavorably both on the authors and the scientific publication system in general. Unfortunately, failures like this one abound in our field and beyond. What we need more of are substantive theoretical discussions, but they keep on getting overshadowed by false or shallow controversies, as well as self-serving public-relations campaigns without much intellectual depth or substance. It is high time we focus on what really matters again. Conceptual progress is at stake.

But let me finish on a more positive note: shortly after the publication of the paper discussed here, another publication came out in *PNAS*, which is similar in spirit, tackling the problem of increasing complexification in general evolutionary processes (Wong et al. 2023). Just like assembly theory, it takes a very broad picture of evolution and “selection,” going far beyond the biological realm of Darwinian evolution by natural selection. In contrast to the paper discussed here, it does not overstate its case, defines its terms precisely (especially when concepts are employed in unusual ways), and frames its discussion in terms of a specific notion of “function.” I have some criticisms regarding these authors’ approach as well, but reading this paper will be a good use of your time. Please engage its ideas with an open mind! They are well worth considering, and may even inspire you to think differently, or reflect on some of the aspects of your own approach to evolution.

## Footnotes

1 The replies to this post on X/Twitter https://twitter.com/Nature/status/1709800557257306572?s=20, present a fairly representative sample of the kinds of reactions the paper generated online.

2 See this thread on X/Twitter, for example: https://x.com/mitomaths/status/1711320060197736918?s=20.

3 A blog post by Hector Zenil (https://hectorzenil.medium.com/test-8f0be54817c4) identifies no less than eight fallacies of assembly theory. Scroll to the section “Original Post” for the actual beginning of the article. A video essay by the same author (https://www.youtube.com/watch?v=078EXZeS8Y0) summarizes these fallacies, and highlights conceptual/methodological limitations, and the pervasive failure by the proponents of assembly theory to acknowledge relevant previous work in the field of complexity science.

4 The original aim was to classify molecules present in an exoplanet atmosphere into those that are likely to be of organic and biological origin. See also Zenil et al (2018).

5 Because of their locally restricted occurrence, the Wikipedia page for Schnitzel hammers only exists in German: https://de.wikipedia.org/wiki/Fleischklopfer.
